# Cone Beam Computed Tomography Assessment of the Maxillary Incisive Canal and Foramen: Considerations of Anatomical Variations When Placing Immediate Implants

**DOI:** 10.1371/journal.pone.0117251

**Published:** 2015-02-13

**Authors:** Samah M. Al-Amery, Phrabhakaran Nambiar, Marhazlinda Jamaludin, Jacob John, Wei Cheong Ngeow

**Affiliations:** 1 Dept. of Diagnostic and Integrated Dental Practice, Faculty of Dentistry, University of Malaya, Kuala Lumpur, Malaysia; 2 Dept. of Community Oral Health and Clinical Prevention, Faculty of Dentistry, University of Malaya, Kuala Lumpur, Malaysia; 3 Dept. of Oro-Maxillofacial Surgical and Medical Sciences, Faculty of Dentistry, University of Malaya, Kuala Lumpur, Malaysia; University of Oulu, FINLAND

## Abstract

**Introduction:**

The maxillary incisive canal connects the roof of the oral cavity with the floor of nasal cavity and has the incisive and nasal foramina respectively at its two opposite ends. Its close proximity with the anterior incisors affects one’s ability to place immediate implants in ideal position.

**Objective:**

To avoid causing complication, variations in their dimensions were studied.

**Material and Methods:**

Images of ninety Mongoloids patients examined with i-CAT Cone Beam Computed Tomography were included. The sizes of the nasopalatine foramen, the incisive canal and foramen, and anterior maxillary bone thickness were measured. The direction and course of the canals were assessed.

**Results:**

The mean labiopalatal and mesiodistal measurements of the incisive foramen were 2.80mm and 3.49 mm respectively, while the labiopalatal width of the nasal foramen was 6.06mm. The incisive canal was 16.33mm long and 3.85 mm wide. The anterior maxillary bone has an average thickness of 7.63 mm. The dimensions of the incisive foramen and incisive canal, and anterior maxillary bone thickness demonstrated gender differences with males showing greater values. The anterior maxillary bone thickness was affected by age but this difference was not observed in canal dimensions. The majority of subjects have a funnel shape-like incisive canal with the broader opening located at its superior. They seem to have a longer slanted-curve canal with one channel at its middle portion and a narrower incisive foramen opening than those reported elsewhere.

**Conclusions:**

This study found that gender is an important factor that affected the characteristics of the IC and the amount of bone anterior to it. Male generally had bigger IC and thicker anterior bone. In addition, the anterior maxillary bone thickness was affected by aging, where it becomes thinner with increased age even though the subjects were fully dentate.

## Introduction

The maxillary incisive papilla is an important landmark in prosthetic dentistry for setting up teeth as it helps to ensure that the arrangement and alignment conforms to the midline of the anterior maxillary ridge [1]. It is also an important landmark when it comes to the administration of local anesthesia in the anterior palate [2]. This is because the maxillary incisive foramen (IF) that is located beneath it marks the exit of the nasopalatine (incisive) nerve that supplies the palatal region of the anterior palate. This foramen is the inferior opening for a passage (the maxillary incisive canal) that connects the oral cavity to the nasal cavity [3].

The IF is an oval-shaped opening that faces the posteroinferior side of the palate. It has a diameter of 2 mm to 1 cm [4–7], with a position that varies from just above the crest of the alveolar ridge to the level of the apices of the central incisors [8].

The maxillary incisive canal (IC) is a Y-shaped passage that is between 4 and 26mm in length, depending on the surrounding maxillary bone height [7, 9]. It develops from the fusion of the right and left IC respectively with the anterior palatine canal to form the common IC [8]. It is located about 12–15 mm from the anterior nasal spine, usually closer to the nasal septum [9]. It connects the roof of the oral cavity with the floor of nasal cavity [8]. The IF and the incisive fossa form the inferior part of this canal while superiorly, the nasal septum in the nasal floor divides the opening into 2 foramina, namely the nasopalatine foramen or the foramen of Stensen [10,11]. Two accessory minor openings, termed the foramina of Scarpa are sometimes seen. These additional canals may also transmit the nasopalatine nerve [7]. Nasal foramen (NF) is the collective term that is usually used to describe the nasal openings located on the nasal floor. In addition to nerve bundles, the naso-(spheno) palatine artery also shares a course along this canal to supply the oral cavity [8]. The maximum width and standard deviation of the NF was reported to be 4.9 (1.2) mm [7].

In general, no serious clinical complication has been reported to result from surgical intrusion to the content of the IC [12], although a case hematoma formation 1 week after surgical removal of an impacted supernumerary tooth in the maxilla has been reported [13]. Neurosensory changes that usually happen following surgical transection of the nasapalatine nerve is felt the most during the first week, but return to normal within a month [12].

However, there is concern over the long term outcome of implants when being in contact with the content of the IC. Two studies suggested that more care should be taken with females and younger patients during the immediate implant placement due to natural root proximity to the IC at the mid-root level of the maxillary central incisor [14, 15]. Taking into consideration future apicolingual resorption, surgeons tend to place implants more palatally. This approach again increases the risk of it encroaching into the IC. As a consequence, there may be a loss of osseointegration at a wall of the endosseous implant [16]. To overcome this problem, Artzi et al reported a novel surgical approach using a configurated cortico-cancellous block graft core to fit the foramen, while its soft tissue content was pushed back posteriorly [17]. This process ensures that solid bone is present around the entire length of the endosseous implant. Nevertheless, preoperative assessment using conventional radiographs may not show the exact anatomy of the IC.

A thorough understanding of variation in sizes of these structures is very important to avoid complications during and following implant insertion. It is therefore, the aim of this study to determine the norms and variations in the location and dimension of the IF and IC in 2 Mongoloid groups, namely the Chinese and Malays. The objective of this study was to determine the variations in (1) the IF size (2) NF size (3) IC length and width (4) the number of channels at the middle portion of the IC and (5) the direction and course of the IC. In addition, the amount of bone present labial to the Nasopalatine (NP) canal was measured. For the purpose of this study, this bone width was termed anterior maxillary bone thickness. The effect of gender and age of the subjects toward these findings were also analyzed.

## Materials and Methods

The study was approved by the Faculty of Dentistry University of Malaya Medical Ethics Committee (IRB approval no.DF DP 1302/0013[P]). The board of ethics was aware that this was a retrospective study and the facts that this study was undertaken using patients’ data / records / radiographs. As this is a teaching institution, all patients seeking treatment at the Faculty of Dentistry are informed and verbal consent taken for all forms of their records to be used for teaching and/or research purposes, with the assurance that their identity will remain anonymous.

### Image selection

One hundred consecutive cone beam computed tomography (CBCT) images recorded with i-CAT imaging system (Imaging Sciences International, Inc. Hatfield, USA), taken at the Oral & Maxillofacial Radiology Division, Faculty of Dentistry, University of Malaya were selected for this study. All images were taken retrospectively but following a standardized protocol for patient positioning, exposure parameter (120 KvP, 3–7 mA, 20 sec) and image acquisition at 0.3 mm voxel size by the same radiographer. These images were reconstructed from the CBCT data using proprietary i-CAT image reconstruction software. For this study, all the patient images were anonymized and de-identified prior to analysis. Patients with suspected pathological lesions in the anterior maxillary region and images of low quality were excluded from this study. A total of 6 records were excluded at this stage.

### Methods

**Fig 1:**
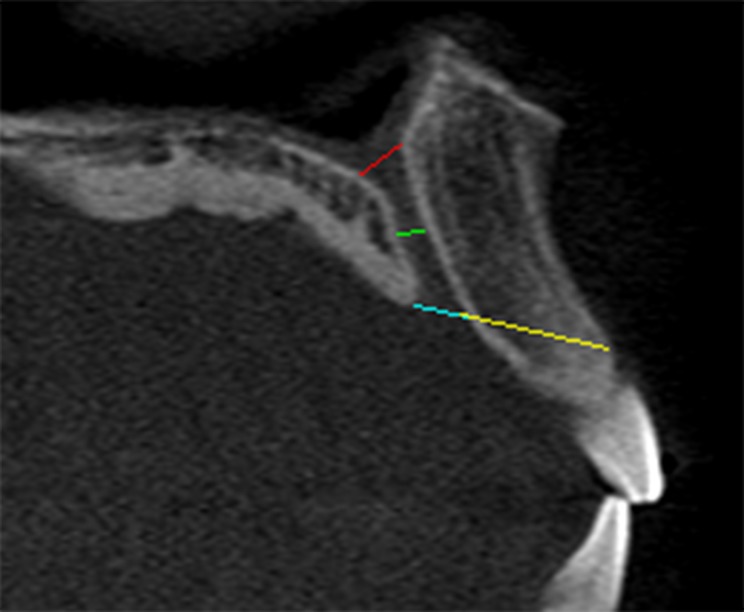
Measurement of incisive foramen (blue line), nasal foramen (red line), incisive diameter (green line), and the incisive foramen location (yellow line).

**Fig 2:**
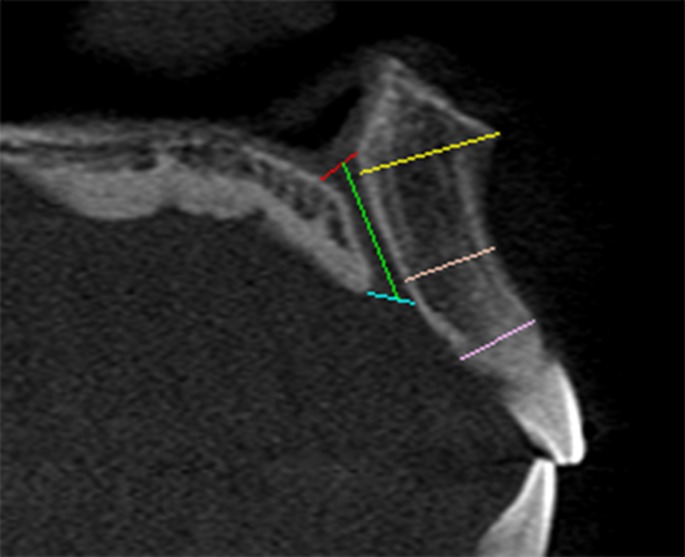
Measurement of the incisive canal length (green line) and anterior maxillary bone thickness at 3 levels (yellow, orange & pink lines).

In general, various measurements were made to determine the dimensions of the IC and corresponding anterior maxillary bone thickness (Figs. 1 and 2). The number of channels at the middle portion of the IC was counted and the course and direction of the IC was determined based on the criteria described by Song et al [6].

Figs. 1 and 2 illustrate various points of measurement at a sagittal-cross section of a CBCT image. This image was obtained using the MPR screen at the middle of the IC. The diameter of the NF was measured at the nasal entrance of the IC while the diameter of the IF was measured at the oral entrance of the canal (Fig. 1). The canal diameter was measured at the midpoint between these two levels (Fig. 1). Using two other images captured at both right and left sides of the IC, the length of the NP canal was measured as a distance from the nasal opening to the palatal opening (Fig. 2).

The anterior maxillary bone thickness (labial to the canal) was assessed at three levels—at level of the nasal spine, at the level of the most anteroinferior point of the cortical plate of maxilla and at the midpoint between these two levels. The measurement was performed as a perpendicular distance from the outer canal wall to the outer cortical plate of anterior maxillary bone (Fig. 2). An average was calculated based on these three measurements, similar to that reported by Mraiwa et al [7]. Lastly, the location of IF was determined as a distance between the IF and the most anteroinferior point of the cortical plate of the labial bone of the maxilla (Fig. 1), as described by Liang et al [18].

**Fig 3:**
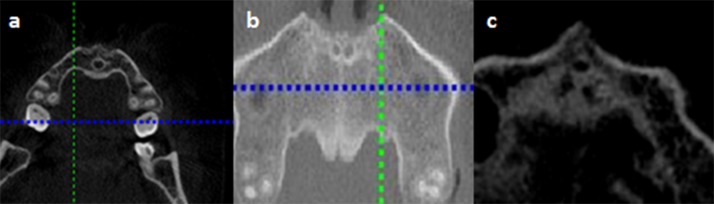
Representative images showing different number of channels seen at mid incisive canal.

The number of channels in the middle portion of the canal was determined by using an axial cross-section view that showed the middle portion of the NP canal (Fig. 3). The term “channel” here refers to separate small canaliculi within the NP canal and is adopted from the term used by Song t al [6].

**Fig 4:**
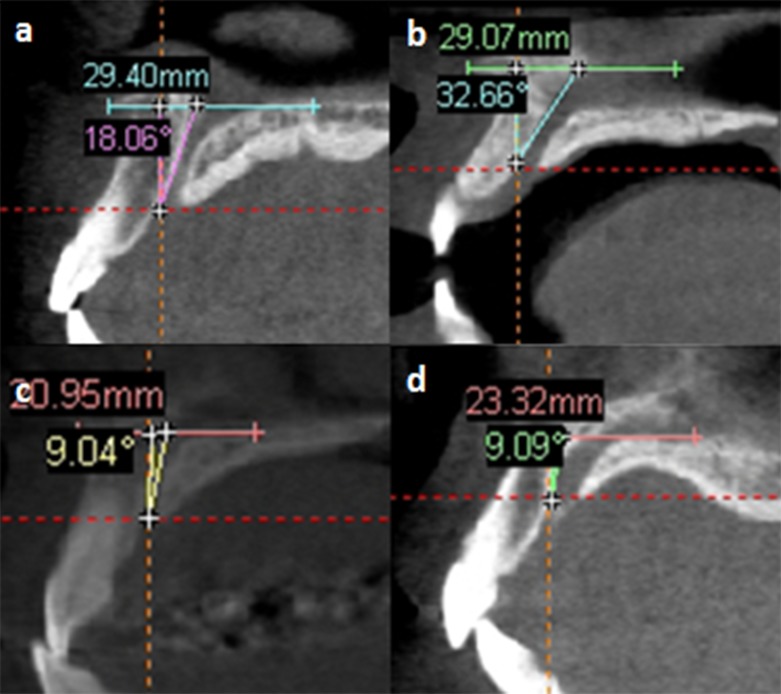
Representative images showing different classifications of the direction and course of the incisive canal.

The IC was then classified according to their sagittally viewed direction in relationship to the nasal floor which was regarded as the horizontal plane. Based on a vertical line drawn perpendicular to the horizontal plane, IC which changed course by more than 10° from vertical was regarded to be “slanted”. Subsequently the course of the IC was classified as either being straight or curved (Fig. 4), as described by Song et al [6]. The Simplant software version 13 was used for this purpose.

**Fig 5:**
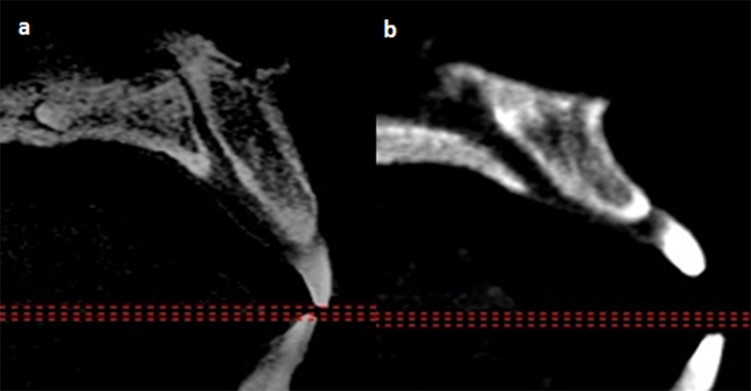
Sagittal view shows the typical appearance of a (a) flat and (b) concave alveolar maxillary bone labial to the incisive canal.

Using the same screen, the shape of the anterior maxillary bone labial to the IC was classified as either being (a) straight (b) concave or (c) convex. Examples of the straight and concave appearance are shown in Fig. 5.

The collection of data was done by the first author. Reliability of the measurements was performed by re-evaluating randomly 10% of samples twice after one week interval and without any knowledge of previous measurements. Intra-class correlation coefficients of 0.97 were achieved showing reproducibility of the evaluation of the categorical data, namely the number of channels seen and the shape and of the IC and the anterior maxillary bone labial to it was good. A kappa figure of 0.92 was achieved when comparing the numerical data, again showing good reliability of results obtained.

### Statistical analysis

Data was entered and analysed by using SPSS 12.01 for Windows (SPSS Inc., Chicago, USA). Descriptive statistic was employed where appropriate. Mean and standard deviation was obtained for all measurements. Differences in measurements between gender were compared using independent *t-test*. Differences in the location of the IF in patients of various age groups were analyzed using analysis of variance (ANOVA). Corresponding non-parametric test (Mann-Whitney U test or Kruskal-wallis test) were used if the distribution of the data was not equal or when the number of the samples was small. In addition, chi-square test was used to compare the differences in the number of channel and the course and direction of the IC. The significance level for all the tests was set at *P < 0.05*.

## Results

Ninety images of 46 males and 44 females between the ages of 15 to 75 years were included into this study. Images of four edentulous persons were excluded from the analysis as it was feared that it may have a negative effect on the morphology of the IC. Twenty of subjects whose images were used in the study were aged between 15–25 years old, 17 were 26–35 years, while 19 each were in the 36–45, 46–55 and 56–75 years old category respectively. Their gender distribution in each age group was almost at 1:1 ratio (range 0.9 to 1.13).

On average, the mean labiopalatal and mesiodistal sizes of the IF was 2.80mm and 3.49mm, respectively (Table 1). This means that it has an elliptical shape with the wider part closer to the adjacent central incisors. There was a significant gender difference in the labiopalatal width (Independent *t-test*; *P* = 0.003) where the male subjects had larger foramen (3.03mm) than female subjects (2.56 mm), but no such significant difference was detected with regards to the mesiodistal width. Female subjects also presented with a wider range in the labiopalatal size (0.95–5.10mm) when compared to the mesiodistal size (1.28–4.80mm).

**Table 1:** The mean dimensional measurement of the incisive and nasal foramina.

Landmarks	Overall average size (SD) & range in mm	Gender		*P* value
		Average size (SD) & range in mm		
		Male (n = 46)	Female (n = 44)	
Incisive foramen (IF)					
Labiopalatal width	2.80 (0.82)	3.05 (0.77)	2.56 (0.80)	0.003*
		[0.95–5.10]	[1.28–4.80]		[0.95–5.10]
	Mesiodistal width	3.49 (1.00)	3.55 (1.05)	3.44 (0.96)	0.624
		[1.50–6.60]	[1.80–6.60]	[1.50–5.70]	
	Distance to labial alveolus	12.05 (3.07)	11.92 (2.76)	12.19 (3.38)	0.686
		[3.45–18.45]	[4.9–16.27]	[3.45–18.45]	
					
Nasal foramen (NF)					
	Labiopalatal width	6.06 (3.00)	6.54 (3.18)	5.56 (2.72)	1.180
		[1.26–16.03]	[1.26–16.03]	[2.42–15.60]	
	Width on right NF	6.03 (2.97)	6.76 (3.31)	6.76 (3.31)	0.017*
		[1.53–15.49]	[1.62–15.49]	[1.62–15.49]	
	Width on left NF	5.93 (3.04)	6.45 (3.32)	5.39 (2.63)	0.098
		[0.90–16.57]	[0.90–16.57]	[1.70–13.12]	

* Independent *t-test*.

**Fig 6:**
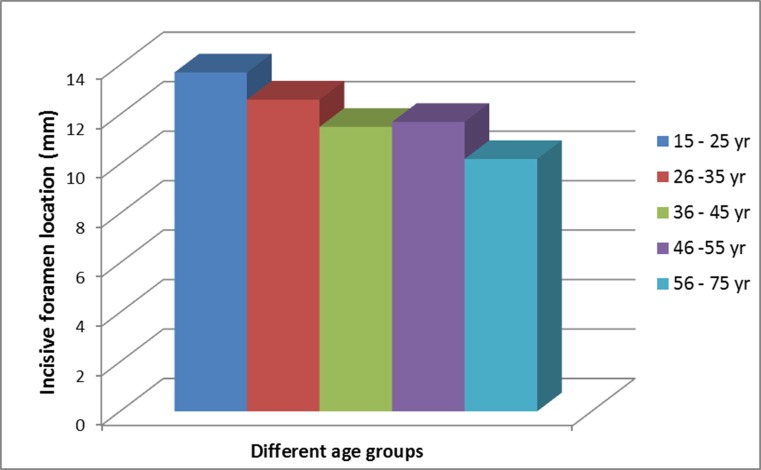
Differences in the distance from the incisive foramen to the most anteroinferior point of the cortical plate of labial bone in subjects of different age groups.

The IF was located 12.05 mm away from the most anteroinferior point of the cortical plate of the labial bone of the maxilla (Table 1). However, there was a wide variation in the ranges of this distance, from 3.45 to 18.45 mm. There was no significant gender influence with regards to this distance, with the average for males being 11.92mm (range 4.97–10.27mm) and the average for females being 12.19 mm (range 3.45–18.45mm). However, one female subject presented with a minimal distance that was almost one-third of the average distance. The location of the IF was affected significantly by advancing age (ANOVA test; *P* = 0.008), where the canal becomes closer to the most anteroinferior point of the cortical plate of the labial bone in older subjects (Fig. 6). Subjects of 15–25 years age group presented with the IF almost 14 mm away from the anteroinferior point of the cortical plate, but this distance was just slightly above 10 mm for those aged more than 55 years. The difference between this two ends of different age-groups is statistically significant (Post Hoc Tukey; *P* = 0.004). Further analysis of dividing the subjects to those below and above 45 years old found that the distance in the former group (12.66 mm) in average, is 1.6 mm more than the latter group (11.06 mm). This difference is statistically significant (Independent *t-test*; *P* = 0.016).

As for the NF, it showed a larger dimension with a mean labiopalatal width of 6.06 mm which is more than double the width of the IF. The NF however, presented with a wide range of widths, from a minimum of 1.26 to as wide as 16.03mm. Further analysis showed that there was no significant difference between mesiodistal widths of the right and left NF (Independent *t-test*; *P* = 1.14). However, there was gender influence on the size of the right NF (Independent *t-test*; *P* < 0.001), with the mesiodistal width for males (6.70mm) being significantly larger than for the females (5.28mm).

The IC has an average width of 3.85mm (Table 2). The canal of male subjects was found to be significantly larger than the female subjects (Independent *t-test*; *P* = 0.015, and when this result was analyzed together with those found for the IF and NF, it suggests that there was gender difference in the size of the IC beginning from its inferior opening until its mid-length.

**Table 2:** The mean dimensional measurement of the maxillary incisive canal.

Incisive canal		Overall average size (SD) & range in mm	Gender		*P* value
			Average size (SD) & range in mm		
			Male (n = 46)	Female (n = 44)	
IC width		3.85 (1.32)	4.17 (1.45)	3.51 (1.09)	0.015*
		[1.50–7.20]	[1.51–7.20]	[1.50–6.14]	
					
Overall IC length		16.33 (4.43)	17.96 (3.96)	17.49 (4.18)	<0.001*
		[4.51–30.24]	[10.64–30.24]	[10.26–31.71]	
	Right IC length	16.66 (4.52)	18.44 (4.12)	14.80 (4.20)	<0.001*
		[4.84–28.77]	[9.84–28.77]	[4.84–23.24]	
	Left IC length	16.00 (4.80)	17.49 (4.18)	14.45 (4.95)	0.002*
		[4.18–31.71]	[10.26–31.71]	[4.18–31.70]	

* Independent *t-test*.

The IC was 16.33mm in average length, with the right canal being slightly longer than the left canal. There was a wide range in the lengths of this canal (4.51–30.24mm). This variation is greater in females who presented with differences in length that is almost six-folds between the shortest and longest canal (4.15–23.97 mm). Males, in contrast presented with a three-fold difference between the longest and shortest canal of 10.64 and 30.24 mm respectively. Further analysis showed that the IC length was significantly longer in male subjects (Independent *t-test*; *P* < 0.001). Interestingly, the age of the patients did not influence the IC length and width (ANOVA test; *P*>0.05).

A majority of the IC presented with 1 channel (64.4%) with the remaining 34.4% having 2 channels. Only 1 IC had 3 channels. The gender of the subjects did not correlate with the number of channels present (chi-square test; *P* = 0.304). There was also no correlation between the length and width of the IC with the number of channels present.

There was a wide variation in the direction and course of ICs when examined in the sagittal view. The slanted type dominated the direction of IC. The most commonly found IC direction and course was the slanted-curve type, accounting for 65.2% in males and 63.6% in female (Table 3). However, there was no significant difference between gender in its direction and course (chi-square test; *P* = 0.382).

**Table 3:** Distribution of the different directions and courses of the ICs in males and females.

Direction & course	Gender n (%)		Overall
	Male	Female	
Slanted-curve	30 (65.2%)	28 (63.6%)	58 (64.4%)
Slanted-straight	15 (32.6%)	14 (31.8%)	29 (32.2%)
Vertical-straight	0 (0%)	0 (0%)	1 (1.1%)
Vertical-curve	0 (0%)	2 (4.5%)	2 (2.1%)
Total	46 (100%)	44 (100%)	90 (100%)

**Fig 7:**
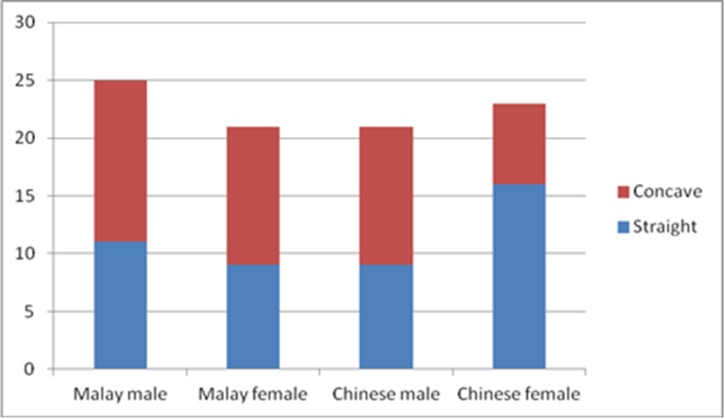
Distribution showing alveolar bone shape anterior to the incisive canal in subjects of different gender and ethnicity.

There was a wide range in anterior maxillary bone thickness. The anterior maxillary bone was the thickest at the nasal spine level (10.75mm), and was the narrowest at lower labial alveolus (5.7mm), giving it a tapering appearance superior-inferiorly. Objective study of the shape of the anterior labial bone anterior to the IC found that they were equally distributed between straight and concave type. None of the bone was convex in shape. Their distribution is shown in Fig. 7. Ethnicity and gender of the subjects did not affect the shape of this bone (Chi-square test: P>0.05).

The average anterior maxillary bone thickness was calculated by obtaining an average of measurements made at three different levels; this was found to be 7.63 mm (Table 4). On the whole, the labial bone thickness in males (8.16mm; range = 4.20–12.18 mm) was significantly larger than in the females (7.08mm; range = 3.92–9.84 mm) (Independent *t-test*; *P* < 0.001), at all the levels of measurement.

**Table 4:** Anterior maxillary bone thickness.

Level		Mean (SD); range in mm	*P* value
Nasal spine			
	Male	11.48 (2.14); 6.64–16.80	0.001*
	Female	10.00 (1.88); 6.35–14.09	
	Average	10.75 (2.14); 6.35–16.80	
			
Mid Incisive canal			
	Male	6.73 (1.90); 2.72–10.60	0.002*
	Female	5.86 (1.55); 1.58–8.22	
	Average	6.31 (1.78); 1.58–10.60	
			
Lower labial alveolus			
	Male	6.19 (2.06); 1.05–9.41	0.055
	Female	5.36 (1.99); 1.53–8.99	
	Average	5.78 (2.06); 1.05–9.41	
			
Overall average		7.63 (1.61); 3.92–12.18	

* Independent *t-test*.

**Fig 8:**
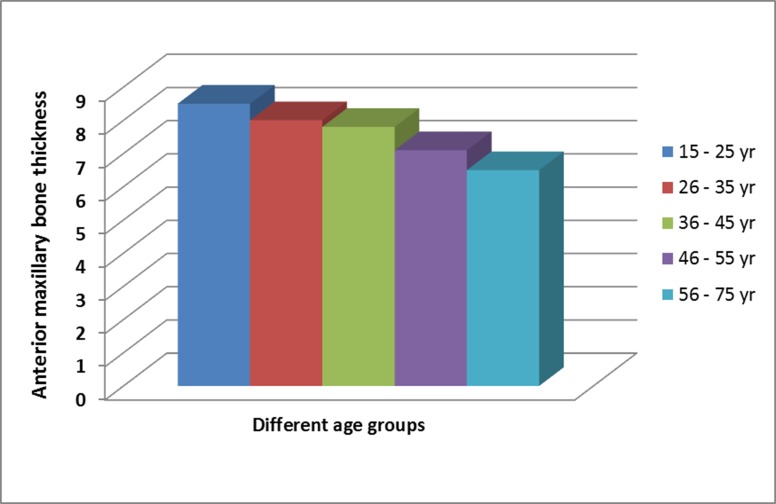
The reduction in anterior maxillary bone thickness with an increase of age.

Age affected significantly the average anterior maxillary bone thickness, whereby it was noted to be thicker in young patients e.g. 8.54 mm in 15–25 years, but reduced 24% to 6.49mm when compared to subjects above 55years (Fig. 8; *ANOVA*; *P* < 0.001). When the data was further analyzed by grouping the subjects into those ≤ 45 years and > 45 years, it was found that the labial bone thickness at the nasal spine was almost equal, but the older patients had significantly narrower anterior maxillary bone thickness at lower labial alveolus and the mid IC level (Independent *t-test*; *P* < 0.001; Table 5). There is a significant positive correlation between the overall location of the IF with the anterior maxillary bone thickness (Pearson’s correlation 0.581; *P* < 0.001).

**Table 5:** Differences in average anterior maxillary bone thickness between those aged above and below 45 years.

Level	Age	Mean (SD) mm	*P* value
Nasal spine	≤ 45 years	10.96 (2.27)	0.227
	> 45 years	10.35 (1.87)	
			
Mid Incisive canal	≤ 45 years	6.93 (1.61)	<0.001
	> 45 years	5.28 (1.57)	
			
Lower labial alveolus	≤ 45 years	6.43 (1.79)	<0.001
	> 45 years	4.71 (2.05)	
			
Average bone thickness	≤ 45 years	8.13 (1.54)	<0.001
	> 45 years	6.80 (1.38)	

* Independent *t-test*.

## Discussion

Implant restoration of the upper incisors is a challenging task due to its aesthetic and functional requirement, amid the limitation that results from the resorption of alveolar bone following tooth extraction, and a variation in the dimension and location of the IC and the IF [19,20]. The former has been addressed by many researchers through various bone graft technique and materials used [13]. However, anatomic limitation arising from the close proximity of the IF and large IC limits one’s ability to immediately place implants in the ideal position.

Although the IC is discussed in the literature, there are only a handful of studies describing the morphology and morphometric variations of this canal [7,15,18,21–26] of which only three involved Asian subjects[6,13,27]. Most other studies focused on IC pathology and their management [28–30]. As such, the authors feel that it is timely to further evaluate the variations in dimensions of IF and IC of two Mongoloid groups, namely the Chinese and Malay.

This current study shows that the IC presents with a wide variation of dimension and course and direction that is not affected by age, but their sizes were influenced by the gender of the subject. This finding is in agreement to that reported by several authors [13, 15, 24, 25]. Although the average dimensions may not be much different from those reported in the literatures (*see below*) their specific variations should be taken into account when placing endosseous implant in the anterior maxilla. The following parts of this section discuss the norm and variations found.

The mean labiopalatal and mesiodistal widths of the IF were 2.80mm and 3.49mm respectively. This is close to the average of 2.90mm reported for the Caucasians/Arabs [26] and 3.49mm for the Koreans [27] on the same parameters measured. However, it is lower than the 3.8mm size for labiopalatal width and 3.7mm for mesiodistal width reported for the Japanese [13]. Further comparison shows that even larger diameter (4.45–4.6 mm) has been observed in Caucasians [7,24]. The difference in the size of IF between male and female has been inconclusive. Mraiwa et al [7], Bornstein et al [24] and Güncü et al [25] reported that male had significantly larger mean canal diameter as compared to female. The authors found that gender difference was only true for the labiopalatal width, but not applicable to the mesiodistal width. We are unable to explain this finding.

In comparison to the IF, the NF has a wider size of 6.06mm. This width of NF is larger when compared to the Caucasians’ and Koreans’ who only presented with a mean diameter of 3.49–4.9mm [7,24,27]. Although the size of the NF in male patients was bigger than the females’, this difference was not statistically significant. This finding concurs with that reported by Mraiwa et al [7], but is in contrast to that reported by Güncü et al [25].

The mean IC width of 3.85mm was slightly larger than those reported by Liang et al [18], who reported variation between 3.3 mm in cadavers to 3.6 mm when examining different samples using CT scans. This measurement is however larger than those reported in dentate Japanese, whereby the labiopalatal width was 2.8 mm and the mesiodistal width was 3.3 mm [13]. The narrowest canal diameter was 1.5mm and the widest was 7.2mm. This range is close to that reported by Song et al [6] and is within the threshold of 1cm, where any size bigger is suspected to have the presence of a pathology. The IC was significantly larger in male than female, a finding that concurs with those reported by several other researchers [18, 25, 31, 32]. However, compared to the current finding, Güncü et al [25] reported smaller mean canal diameter of 2.79mm in male, and 2.4 mm in female.

Taken together, the measurements obtained for the 3 diameters of the IC suggested an appearance of a funnel shape like passage between the superior (wider NF opening), middle (intermediate IC width) and inferior (narrower IF opening), similar to that reported by Mraiwa et al [7] and Kim et al [27]; but this finding is in contrast to that reported by Asaumi et al [13] who found the opening to be broader at the oral cavity side. Mardinger et al [15] and Tözüm et al [26] on the other hand, found the middle part of the canal to be the narrowest.

The mean length of IC has been reported to range between 8.1mm and 11.96mm in Caucasians [7,15,18,24–26]. In contrast, Song et al [6] reported longer mean length of 12.0mm and 15.87mm respectively in Koreans. In this study, the IC was found to have mean length of 16.33mm which is close to that reported by Kim et al [27] who also performed their study using CBCT. The IC has been reported to be 1.32–1.57mm significantly longer in male than female Caucasians [24,25]. The current study however, found an even higher difference of 3.27mm. One unique feature is noticeable when the findings for the IF and NF above are combined with this IC length. The Mongoloids in this study seem to have a longer IC with narrower IF opening similar to that reported by Kim et al [27] in Koreans, compared to literatures describing the Caucasians as having shorter canal with wider IF opening.

Several authors described the effect of the age on the length and width of the IC [13,15,18,24,33]. They found that with aging the NP canal diameter increases due to the bone resorption. At the same time, the IC length decreases. However, this study did not find any significant difference in the canal dimensions in patients of different age-groups. This is because there were only four edentulous patients included into this study. Such an observation is consistent to the dimensional stability reported for the fully dentate control group in the study by Mardinger et al [15]. This finding suggests that the presence of teeth ensure stability in the size and length of the IC.

In a cross-sectional study among Koreans, it was found that 87% of IC at the side of nasal fossa have one channel, 10.4% have two channels, and 2.6% have three channels, but these canals merged into one canal in the middle portion of palate [27]. Similar features of multiple channels, with a maximum of 4 channels at the NF have also been reported in Caucasians [7,18]. In contrast, this study found that multiple channels were present even at the middle portion of the palate, a finding similar to that reported by Song et al [6]. A majority of the IC presented with 1 channel (63.8%) with the remaining being mainly 2 channels (35.1%). Only 1 IC had 3 channels. In comparison, Song et al [6] reported a lower percentage of patients presenting with 1 channel (42.9%) but higher percentage of patients presenting with 3 channels (25.0%). They also reported finding 8.9% patients with 4 channels, a finding that was not observed in this study.

The majority of the Mongoloid population in this study have slanted-curve canal with one channel at the middle portion of the IC. Although this result was in agreement with Song et al [6] with regard to the number of the channels, the direction and course of their IC was however, mainly of the vertical-straight type.

The funnel shaped IC is described as having a Y-shape because it is made up of at least two nasal orifices on each side of nasal septum that forms a right and left canals which eventually unite to form a common canal in the incisive fossa region. Comparisons were done to get more information regarding their length and as a result of, the authors can safely say that the right canal is longer than the left canal in both males and females. The reason for this developmental variation is still unknown.

Equally important for immediate implant placement is the anterior maxillary bone thickness. Similar to that reported by several researchers [13,24–26], the bony dimensions demonstrated an increasing thickness from crestal to apical measurements. The basal bone does not change its shape significantly [34], and the current study corresponds with this finding, whereby the anterior maxillary bone thickness at the anterior nasal spine area was not different between the young (10.96mm) and the old (10.35mm) subjects. This thickness is however slightly lower than the average of 12–15 mm reported in Caucasians [9] but is higher than that reported for the Japanese [13]. The mean thickness of 7.54mm as obtained by averaging the measurements at 3 levels was close to the average reported by Mraiwa et al [7] and Asaumi et al [13] but lower to that reported for the Chinese [35]. The range of 3.92–12.18mm was also not much different from those reported by Mraiwa et al [7], and because of this, the precautionary advice by Mraiwa et al [7] on the need to carefully examine each variation in patients preoperatively must be followed for Mongoloid subjects too.

This study found that the anterior maxillary bone thickness in male was more than female, a finding in agreement with those reported by several researchers [18,24–26]. Such a finding indicates that female patients need more precautions during surgical procedures. Furthermore, researchers [15,17,26] reported that aging causes anterior maxillary bone thickness to reduce and some relate this to the fact that these patients most likely lose the anterior teeth as their age increases. An unusual finding in this study was that even in fully dentate patients, the anterior bone width became less (a 24% reduction) as the age of the subjects increase. The cause is unknown even though it may be related to bone remodeling and structural loss with advancing ages.

Although there is no significant difference in the location of the IF between males and females, in general males exhibited closer position to the most anterioinferior point of the cortical plate of the labial bone of the maxilla (mean = 11.92mm) while for female, the mean was 12.19mm. Both measurements were higher than those reported by Liang et al [18], who found an average of 10.6 mm in their dentate group. This current study revealed a possible effect of aging on the location of the IF when the anterior maxillary bone thickness and the distance to the most anteroinferior point of the cortical plate of labial bone become reduced with increase in age. These changes will make the procedure of dental implantation become more complex for aged patients. In such cases, this problem can be overcome by surgical intervention where the soft tissue contents in the canal is either curreted or pushed back, and the IF obturated by bone graft [17,36,37]. In fact, Peñarrocha et al [37] even used the IC as an anatomic buttress for implant placement.

In this study CBCT and the SimPlant interactive software were used to visualize the anterior maxilla for cross-sectional imaging. This technique confirms the benefit for replacing multi-detector CT scans in order to obtain accurate diagnosis and evaluation of structures in this area. CBCT machines generate less radiation with high-quality images sufficient for invasive procedures such as implant insertion and also bone grafting [13,24].

The clinical implication of this study reveals the fact that there is great variability in the sizes of the nasopalatine foramen, the incisive canal and foramen, and anterior maxillary bone thickness, even within subjects of the same Mongoloid origin. These differences appear to be gender and age related rather than of ethnic difference. Nevertheless, this study provides a detailed anatomic location of the IC and the sizes of the adjacent structures that shall become a useful reference in centers where CBCT is not available. It is recommended that clinicians make use of CBCT to overcome the shortcomings observed in conventional radiography, where possible.

## Conclusion

This study clearly showed wide variations in the IF widths, IC dimensions and anterior maxillary bone thickness with significant difference between gender, where male patients showed larger and longer IC dimensions. This anatomical variability in the dimensions may be clinically important during surgical procedures especially in immediate implant placement. The findings from this study suggest that gender is an important factor that can affect the characteristics of the IC and the amount of bone anterior to it. In addition, the anterior maxillary bone thickness and the location of the IF are affected by aging. Lastly, Chinese and Malay subjects appear to have a funnel shape-like IC with the broader opening located at its superior. They seem to have a longer slanted-curve IC with one channel at its middle portion and a narrower IF opening than those reported in other population.

## Supporting Information

S1 Datasheet Combined file of supporting tables. Table S1: Measurements of the incisive canal structure, number of channel at the middle of the canal, course and direction of the canal and the anterior bone thickness in Malay males. Table S2: Measurements of the incisive canal structure, number of channel at the middle of the canal, course and direction of the canal and the anterior bone thickness in Malay females. Table S3: Measurements of the incisive canal structure, number of channel at the middle of the canal, course and direction of the canal and the anterior bone thickness in Chinese males. Table S4: Measurements of the incisive canal structure, number of channel at the middle of the canal, course and direction of the canal and the anterior bone thickness in Chinese females.
(DOCX)
