# Supplementary material for: Cone Beam Computed Tomography Assessment of the Maxillary Incisive Canal and Foramen: Considerations of Anatomical Variations When Placing Immediate Implants
Source: PLoS One. 2015 Feb 13;10(2):e0117251. doi: 10.1371/journal.pone.0117251 (PMC4332502; doi:10.1371/journal.pone.0117251)
Supplement: S1 Datasheet — Table S1: Measurements of the incisive canal structure, number of channel at the middle of the canal, course and direction of the canal and the anterior bone thickness in Malay males. Table S2: Measurements of the incisive canal structure, number of channel at the middle of the canal, course and direction of the canal and the anterior bone thickness in Malay females. Table S3: Measurements of the incisive canal structure, number of channel at the middle of the canal, course and direction of the canal and the anterior bone thickness in Chinese males. Table S4: Measurements of the incisive canal structure, number of channel at the middle of the canal, course and direction of the canal and the anterior bone thickness in Chinese females. (DOCX) [file pone.0117251.s001.docx]

**Table S1. Measurements of the incisive canal structure, number of channel at the middle of the canal, course and direction of the canal and the anterior bone thickness in Malay males.**

| **No.** | **Incisive foramen LPø^a^** | **Incisive Foramen MD ø^b^** | **Nasal foramen ø^c^** | **Right foramen nasal ø** | **Left nasal foramen ø** | **IC length^d^** | **Right IC length** | **Left IC length** | **IC width** | **Bone thickness** | **Incisive foramen location** | **NO. channel** | **Course and direction** |
| --- | --- | --- | --- | --- | --- | --- | --- | --- | --- | --- | --- | --- | --- |
| **1** | 2.32 | 1.8 | 1.26 | 1.62 | 0.9 | 18.4 | 18.68 | 18.13 | 1.51 | 7.69 | 11.63 | 1 | Slanted-curve |
| **2** | 1.97 | 3.6 | 4.2 | 3.58 | 4.83 | 12.56 | 12.9 | 12.23 | 2.69 | 11.86 | 13.34 | 2 | Slanted-curve |
| **3** | 4.04 | 6.6 | 11.63 | 12.9 | 10.36 | 18.67 | 19.31 | 18.03 | 6.81 | 6.7 | 13.55 | 2 | Slanted-straight |
| **4** | 3.24 | 2.7 | 6.38 | 7.92 | 4.85 | 19.79 | 18.56 | 21.03 | 3.63 | 10.44 | 16.11 | 2 | Slanted-curve |
| **5** | 3.1 | 4.4 | 6.96 | 10.63 | 3.3 | 19.61 | 22.15 | 17.08 | 4.52 | 6.34 | 9.55 | 2 | Slanted -curve |
| **6** | 2.7 | 3.3 | 3.38 | 3.35 | 3.42 | 16.3 | 17.06 | 15.54 | 2.99 | 9.99 | 16.07 | 1 | Slanted-curve |
| **7** | 3.3 | 3 | 16.03 | 15.49 | 16.57 | 27.47 | 27.95 | 27 | 7.2 | 6.04 | 10.64 | 1 | Slanted-curve |
| **8** | 2.95 | 3.9 | 4.8 | 4.5 | 5.11 | 20.78 | 21.42 | 20.14 | 3.36 | 9.78 | 15.33 | 1 | Slanted-curve |
| **9** | 3.35 | 3.3 | 12.96 | 12.96 | 12.96 | 15.71 | 16.99 | 14.43 | 6.26 | 9.48 | 14.71 | 2 | Slanted-straight |
| **10** | 2.99 | 3.6 | 5.2 | 5.82 | 4.59 | 17.56 | 16.1 | 19.03 | 3.16 | 7.93 | 14.69 | 1 | Slanted-curve |
| **11** | 3.85 | 3.61 | 5.79 | 6.41 | 5.18 | 19.7 | 18.81 | 20.59 | 3.8 | 7.64 | 10.85 | 1 | Slanted-curve |
| **12** | 3.38 | 2.4 | 4.27 | 4.46 | 4.08 | 19.58 | 19.61 | 19.55 | 2.99 | 10.26 | 13.45 | 1 | Slanted-curve |
| **13** | 2.03 | 3.9 | 7.97 | 8.82 | 7.13 | 23.86 | 24.91 | 22.82 | 6.68 | 8.73 | 11.10 | 1 | Slanted-straight |
| **14** | 3.29 | 3 | 3.31 | 4.46 | 2.16 | 14.28 | 18.3 | 10.26 | 2.7 | 6.64 | 12.61 | 2 | Slanted-straight |
| **15** | 1.72 | 2.1 | 6.24 | 6.44 | 6.04 | 21.45 | 22.07 | 20.83 | 2.87 | 6.24 | 6.3 | 1 | Slanted-curve |
| **16** | 3.07 | 3.6 | 5.17 | 5.76 | 4.58 | 15.65 | 15.97 | 15.34 | 3.57 | 7.24 | 11.59 | 1 | Slanted-curve |
| **17** | 2.5 | 3.3 | 5.83 | 6.07 | 5.6 | 17 | 17.58 | 16.42 | 3.34 | 8.52 | 8.3 | 1 | Slanted-curve |
| **18** | 3.52 | 3.3 | 5.45 | 4.33 | 6.58 | 13.87 | 14.24 | 13.51 | 4.04 | 9.33 | 13.73 | 1 | Slanted-curve |
| **19** | 4.5 | 6.3 | 7.33 | 7.25 | 7.42 | 15.84 | 15.34 | 16.35 | 4.93 | 6.44 | 10.59 | 2 | Slanted-curve |
| **20** | 3.23 | 3.9 | 5.16 | 4.02 | 6.31 | 17.71 | 18.43 | 16.99 | 3.68 | 8.44 | 12.55 | 1 | Slanted-curve |
| **21** | 4.38 | 3 | 2.68 | 2.68 | 2.68 | 16.46 | 17.84 | 15.09 | 2.91 | 6.5 | 10.72 | 1 | Slanted-curve |
| **22** | 3.27 | 4.2 | 4.81 | 5.32 | 4.3 | 14.29 | 15.18 | 13.4 | 3.46 | 5.26 | 8.78 | 1 | Slanted-curve |
| **23** | 4.12 | 5.7 | 11.39 | 11.68 | 11.1 | 12.45 | 9.84 | 15.06 | 7.05 | 7.51 | 12.9 | 1 | Slanted-straight |
| **24** | 3.48 | 3 | 5.07 | 8.08 | 7.13 | 20.08 | 20.16 | 20 | 4.42 | 7.72 | 10.56 | 2 | Slanted-straight |
| **25** | 2.9 | 3.9 | 3.79 | 4.08 | 3.5 | 14.15 | 14.7 | 13.6 | 2.72 | 5.36 | 4.97 | 1 | Slanted-curve |

a. Incisive foramen dimension labio-palatel.

c. Nasal foramen dimension.

b. Incisive foramen dimension mesio-distal

d. Incisive canal length.

**Table S2. Measurements of the incisive canal structure, number of channel at the middle of the canal, course and direction of the canal and the anterior bone thickness in Malay females.**

| **No.** | **Incisive foramen LPø^a^** | **Incisive foramen MD ø^b^** | **Nasal foremen ø^c^** | **Right nasal ø** | **Left nasal ø** | **IC length** | **Right IC length** | **Left IC length** | **IC width** | **Bone thickness** | **Incisive Foramen location** | **No. channel** | **Course and direction** |
| --- | --- | --- | --- | --- | --- | --- | --- | --- | --- | --- | --- | --- | --- |
| **1** | 5.1 | 5.21 | 2.42 | 2.95 | 1.9 | 8.14 | 7.93 | 8.36 | 4.23 | 7.78 | 16.56 | 1 | slanted-curve |
| **2** | 3.17 | 5.4 | 5.06 | 5.03 | 5.1 | 4.51 | 4.84 | 4.18 | 3.73 | 7.49 | 17.72 | 1 | Slanted-straight |
| **3** | 2.43 | 1.5 | 3.9 | 5.13 | 2.68 | 17.14 | 17.78 | 16.51 | 2.64 | 8.92 | 16.42 | 2 | Slanted-curve |
| **4** | 2.82 | 3.3 | 6.22 | 5.51 | 6.93 | 16.12 | 16.15 | 16.1 | 4.18 | 7.42 | 9.99 | 1 | Slanted-curve |
| **5** | 1.38 | 2.7 | 3.52 | 1.53 | 5.51 | 11.5 | 10.76 | 12.24 | 1.99 | 8.84 | 11.19 | 2 | Slanted-straight |
| **6** | 3.1 | 3.01 | 3.66 | 3.42 | 3.91 | 9.49 | 9.12 | 9.87 | 3.1 | 8.29 | 13.65 | 1 | Slanted-curve |
| **7** | 2.27 | 3 | 3.61 | 4.04 | 3.19 | 9.68 | 9.67 | 9.7 | 2.35 | 7.36 | 13.15 | 1 | Slanted-curve |
| **8** | 2.47 | 3 | 3.59 | 3.95 | 3.23 | 17.31 | 16.99 | 17.63 | 2.34 | 7.38 | 13.02 | 1 | Slanted-curve |
| **9** | 2.15 | 3.3 | 4.41 | 4.33 | 4.5 | 9.55 | 9.62 | 9.49 | 2.66 | 7.08 | 11.19 | 2 | Slanted-straight |
| **10** | 2.76 | 3.3 | 2.73 | 3.76 | 1.7 | 12.63 | 12.79 | 12.48 | 2.45 | 6.44 | 11.57 | 1 | Slanted-curve |
| **11** | 2.18 | 3.3 | 3.4 | 3.61 | 3.19 | 9.87 | 9.68 | 10.06 | 2.68 | 6.28 | 7.27 | 1 | Slanted-straight |
| **12** | 1.29 | 2.7 | 3.89 | 4.02 | 3.76 | 10.45 | 10.08 | 10.82 | 2.12 | 9.02 | 18.45 | 1 | Slanted-curve |
| **13** | 2.5 | 3 | 6.67 | 7.57 | 5.77 | 14.66 | 14.9 | 14.43 | 4.12 | 9.54 | 14.38 | 1 | Slanted-straight |
| **14** | 3.08 | 3.6 | 6.02 | 6.91 | 5.13 | 14.58 | 15.15 | 14.01 | 4.45 | 6.06 | 10.88 | 1 | Slanted-curve |
| **15** | 1.96 | 4.2 | 5.46 | 4.95 | 5.98 | 22.72 | 23.24 | 22.2 | 3.07 | 7.89 | 12.53 | 1 | Slanted-curve |
| **16** | 3.14 | 4.5 | 5.68 | 5.47 | 5.89 | 9.8 | 9.58 | 10.02 | 3.72 | 6.09 | 14.35 | 2 | Slanted-curve |
| **17** | 2.57 | 3.6 | 3.98 | 4.97 | 3 | 12.09 | 13.6 | 10.59 | 2.86 | 7.75 | 13.98 | 2 | Slanted-curve |
| **18** | 2.8 | 3.3 | 6.12 | 6.36 | 5.89 | 15.53 | 16.89 | 14.17 | 3.68 | 7.1 | 15.3 | 1 | Slanted-curve |
| **19** | 1.96 | 3.6 | 3.64 | 3.24 | 4.04 | 11.67 | 12.73 | 10.62 | 2.82 | 7.01 | 13.74 | 1 | Slanted-curve |
| **20** | 3.57 | 3.3 | 6.61 | 7.69 | 5.53 | 20 | 20.81 | 19.19 | 4.22 | 5.11 | 8.6 | 1 | Slanted-curve |
| **21** | 3.42 | 4.2 | 3.25 | 3.42 | 3.09 | 14.83 | 15.21 | 14.46 | 3.37 | 6.69 | 9.97 | 1 | Slanted-curve |

a. Incisive foramen dimension labio-palatel.

c. Nasal foramen dimension.

b. Incisive foramen dimension mesio-distal

d. Incisive canal length

**Table S3. Measurements of the incisive canal structure, number of channel at the middle of the canal, course and direction of the canal and the anterior bone thickness in Chinese males.**

| **No.** | **Incisive foramen LPø^a^** | **Incisive foramen MD ø^b^** | **Nasal foremen ø^c^** | **Right nasal ø** | **Left nasal ø** | **IC length** | **Right IC length** | **Left IC length** | **IC width** | **Bone thickness** | **Incisive Foramen location** | **No. channel** | **Course and direction** |
| --- | --- | --- | --- | --- | --- | --- | --- | --- | --- | --- | --- | --- | --- |
| **1** | 4.2 | 2.7 | 4.56 | 4.24 | 4.88 | 14.61 | 14.61 | 14.61 | 3.99 | 8.85 | 12.61 | 1 | Slanted-straight |
| **2** | 2.09 | 3 | 10.01 | 10.03 | 9.99 | 15.85 | 16.32 | 15.38 | 5.22 | 8.59 | 16.27 | 1 | Vertical-straight |
| **3** | 2.92 | 3.6 | 11.69 | 9.42 | 13.97 | 30.24 | 28.77 | 31.71 | 5.65 | 12.18 | 12.67 | 2 | Slanted-curve |
| **4** | 3.01 | 3.3 | 7.21 | 6.98 | 7.45 | 18.51 | 18.57 | 18.45 | 4.62 | 9.79 | 12.5 | 2 | Slanted-curve |
| **5** | 3.12 | 3 | 3.63 | 4.43 | 2.83 | 22.7 | 23.08 | 22.32 | 3.27 | 9.32 | 15.58 | 1 | Slanted-curve |
| **6** | 1.6 | 3 | 4.47 | 3.42 | 5.53 | 13.1 | 10.91 | 15.3 | 2.54 | 8.73 | 14.83 | 1 | Slanted-curve |
| **7** | 4.8 | 6 | 8.94 | 9.02 | 8.86 | 18.83 | 19.48 | 18.19 | 6.2 | 8.92 | 13.7 | 2 | Slanted-curve |
| **8** | 2.68 | 4.5 | 5.45 | 4.84 | 6.07 | 16.81 | 20.68 | 12.94 | 4 | 6.22 | 8.45 | 1 | Slanted-curve |
| **9** | 2.68 | 4.2 | 3.9 | 3.51 | 4.3 | 19.13 | 19.86 | 18.4 | 3.08 | 8.79 | 9.62 | 1 | Slanted-curve |
| **10** | 3.16 | 2.7 | 7.44 | 7.8 | 7.08 | 17.43 | 18.46 | 16.41 | 4.84 | 9.58 | 12.2 | 1 | Slanted-curve |
| **11** | 2.76 | 4.2 | 6.92 | 5.92 | 7.92 | 11.77 | 11.28 | 12.26 | 3.87 | 8.47 | 12.48 | 1 | Slanted-straight |
| **12** | 3.56 | 3 | 7.34 | 7.57 | 7.11 | 18.84 | 19.31 | 18.37 | 4.94 | 9.51 | 14.08 | 1 | Slanted-straight |
| **13** | 1.28 | 2.7 | 6.65 | 8.32 | 4.99 | 18.56 | 20.41 | 16.71 | 3.18 | 7.15 | 8.61 | 3 | Slanted-straight |
| **14** | 2.7 | 2.7 | 9.91 | 12.18 | 7.65 | 22.93 | 23 | 22.86 | 5.49 | 9.81 | 12.33 | 1 | Slanted-curve |
| **15** | 3.58 | 3.6 | 7.47 | 6.66 | 8.28 | 18.41 | 21.03 | 15.79 | 5.49 | 4.2 | 6.17 | 2 | Slanted-straight |
| **16** | 3.76 | 2.4 | 6.26 | 5.82 | 6.71 | 21.53 | 21.43 | 21.63 | 4.67 | 7.75 | 15 | 1 | Slanted-curve |
| **17** | 1.56 | 3 | 3.73 | 3.42 | 4.04 | 10.64 | 10.84 | 10.45 | 2.68 | 8.51 | 13.65 | 2 | Slanted-straight |
| **18** | 3.09 | 4.5 | 14.15 | 14.09 | 14.22 | 18.02 | 18.97 | 17.08 | 7.1 | 9.11 | 14.47 | 1 | Slanted-curve |
| **19** | 2.97 | 4.5 | 7.57 | 7.74 | 7.41 | 24.48 | 24.98 | 23.98 | 4.72 | 8.93 | 11.05 | 1 | Slanted-straight |
| **20** | 2.57 | 2.4 | 2.14 | 1.7 | 2.58 | 14.88 | 15.75 | 14.01 | 2.09 | 6.57 | 9.55 | 1 | Slanted-straight |
| **21** | 2.95 | 2.7 | 4.57 | 5.11 | 4.04 | 15.68 | 16.35 | 15.02 | 3.04 | 6.26 | 8 | 1 | Slanted-straight |

a. Incisive foramen dimension labio-palatel.

c. Nasal foramen dimension.

b. Incisive foramen dimension mesio-distal

d. Incisive canal length

**Table S4. Measurements of the incisive canal structure, number of channel at the middle of the canal, course and direction of the canal and the anterior bone thickness in Chinese females.**

| **No.** | **Incisive foramen LPø^a^** | **Incisive foramen MD ø^b^** | **Nasal foremen ø^c^** | **Right nasal ø** | **Left nasal ø** | **IC length** | **Right IC length** | **Left IC length** | **IC width** | **Bone thickness** | **Incisive Foramen location** | **No. channel** | **Course and direction** |
| --- | --- | --- | --- | --- | --- | --- | --- | --- | --- | --- | --- | --- | --- |
| **1** | 2.69 | 3.60 | 8.97 | 6.44 | 11.50 | 23.97 | 16.24 | 31.7 | 5.22 | 7.95 | 10.96 | 2 | Slanted-curve |
| **2** | 0.95 | 1.5 | 2.55 | 2.16 | 2.95 | 5.93 | 6.07 | 5.79 | 1.50 | 7.83 | 16.27 | 2 | Slanted-straight |
| **3** | 1.43 | 1.8 | 9.85 | 6.58 | 13.12 | 22.05 | 17.47 | 26.63 | 4.51 | 9.84 | 12.92 | 1 | Slanted-straight |
| **4** | 2.38 | 2.4 | 9.03 | 9.81 | 8.25 | 11.42 | 12.08 | 10.77 | 4.3 | 6.67 | 16.36 | 1 | Slanted-straight |
| **5** | 1.6 | 2.7 | 5.92 | 5.66 | 6.18 | 20.04 | 21 | 19.09 | 3.28 | 6.25 | 12.68 | 1 | Slanted-straight |
| **6** | 1.81 | 2.4 | 2.5 | 2.58 | 2.42 | 13.6 | 13.98 | 13.23 | 2.17 | 8.84 | 10.59 | 1 | Slanted-straight |
| **7** | 2 | 2.4 | 7.52 | 7.78 | 7.26 | 15.51 | 18.19 | 12.84 | 4.38 | 7.54 | 14.5 | 2 | Vertical-curve |
| **8** | 1.93 | 4.2 | 5.14 | 2.7 | 7.59 | 15.22 | 14.86 | 15.58 | 3.28 | 5.1 | 3.45 | 1 | Slanted-curve |
| **9** | 2.57 | 3.9 | 5.19 | 5.41 | 4.97 | 19.97 | 20.69 | 19.25 | 3.36 | 8.98 | 16.86 | 2 | Slanted-curve |
| **10** | 1.71 | 3.3 | 3.21 | 4.24 | 2.18 | 17.34 | 18.8 | 15.88 | 2.4 | 7.9 | 10.97 | 2 | Slanted-curve |
| **11** | 3.73 | 5.1 | 3.59 | 4.18 | 3 | 18.83 | 19.48 | 18.18 | 3.4 | 5.41 | 13.1 | 2 | Slanted-curve |
| **12** | 2.75 | 3.6 | 15.6 | 4.26 | 7.16 | 15.6 | 14.42 | 16.78 | 4.03 | 7.16 | 6.83 | 2 | Slanted-straight |
| **13** | 2.27 | 3.9 | 5.43 | 5.1 | 5.77 | 12.23 | 12.62 | 11.84 | 3.93 | 6.2 | 7.39 | 2 | Slanted-curve |
| **14** | 2.28 | 2.1 | 11 | 12.73 | 9.27 | 18.07 | 20.44 | 15.71 | 6.14 | 6.28 | 15.38 | 1 | Vertical-curve |
| **15** | 2.37 | 3.9 | 7.49 | 7.16 | 7.82 | 15.63 | 16.98 | 14.28 | 4.49 | 6.58 | 10.36 | 2 | Slanted-curve |
| **16** | 2.56 | 3 | 3.61 | 2.85 | 4.37 | 16.92 | 17.08 | 16.77 | 2.83 | 6.49 | 11.95 | 2 | Slanted-curve |
| **17** | 2.02 | 3.3 | 5.19 | 4.97 | 5.41 | 16.42 | 15.91 | 16.94 | 2.82 | 6.83 | 12.91 | 1 | Slanted-straight |
| **18** | 2.76 | 3.9 | 6.48 | 6.8 | 6.16 | 12.14 | 13.55 | 10.74 | 3.77 | 3.92 | 4.58 | 1 | Slanted-curve |
| **19** | 3.21 | 4.8 | 9.32 | 7.65 | 11 | 15.72 | 14.99 | 16.45 | 5.89 | 5.65 | 10.78 | 2 | Slanted-straight |
| **20** | 3.5 | 4 | 9.53 | 10.06 | 9 | 13.87 | 14.76 | 12.98 | 5.28 | 4.98 | 8.97 | 2 | Slanted-curve |
| **21** | 1.96 | 2.7 | 2.88 | 2.58 | 3.19 | 17.32 | 18.23 | 16.42 | 2.15 | 8.08 | 15.15 | 1 | Slanted-curve |
| **22** | 4.1 | 5.7 | 7.78 | 9.57 | 6 | 16.26 | 17.65 | 14.87 | 5.44 | 5.66 | 10.78 | 2 | Slanted-curve |
| **23** | 3.43 | 4.2 | 2.83 | 3.09 | 2.58 | 16.88 | 18 | 15.76 | 2.98 | 5.63 | 8.55 | 1 | Slanted-curve |

a. Incisive foramen dimension labio-palatel.

c. Nasal foramen dimension.

b. Incisive foramen dimension mesio-distal

d. Incisive canal length
